# Diagnostic utility of ADA and CBNAAT in tubercular pleural effusion

**DOI:** 10.6026/973206300214484

**Published:** 2025-12-15

**Authors:** N. Patel Ronak Kumar, Rahul Soni, Darshi Rastogi, Pankaj Kumar Gupta, Sachin Parmar

**Affiliations:** 1 Department of Pulmonary Medicine, L.N Medical College & J.K Hospital, Bhopal, Madhya Pradesh, India; 2 Department of Pulmonary Medicine, Birsa Munda Government Medical College, Shahdol, Madhya Pradesh, India; 3 Department of General Medicine, Birsa Munda Government Medical College Shahdol, Madhya Pradesh, India; 4 Department of Community Medicine, V.K.S. Government Medical College, Neemuch, Madhya Pradesh, India

**Keywords:** Tubercular pleural effusion, Adenosine Deaminase (ADA), CBNAAT, diagnostic accuracy, sensitivity

## Abstract

The diagnostic performance of Adenosine Deaminase (ADA) and Cartridge-Based Nucleic Acid Amplification Test (CBNAAT) in diagnosing tubercular pleural effusion (TPE) is of interest. Hence, a study was conducted over 18 months at LN Medical College and JK Hospital, Bhopal. The study involved 108 adult patients with TPE. Data shows that CBNAAT, although highly specific, had limited sensitivity, while ADA ≥40 IU/L demonstrated strong sensitivity. ADA levels were significantly higher in CBNAAT-positive participants, suggesting that combining both tests enhances diagnostic accuracy. This approach can help reduce the need for invasive procedures and improve timely treatment, especially in resource-limited settings. We show the importance of using ADA and CBNAAT together in the diagnosis of TPE.

## Background:

Tuberculous pleural effusion (TPE) is the second-commonest form of extra-pulmonary tuberculosis and accounts for a substantial proportion of exudative pleural effusions, especially in high-burden countries [1]. Definitive diagnosis rests on isolation of *Mycobacterium tuberculosis* from pleural fluid or pleural tissue; however, culture yields are low because the disease is paucibacillary, while thoracoscopic or image-guided biopsies, though more sensitive, are invasive, costly and not universally available [2]. Consequently, clinicians rely on surrogate biomarkers such as pleural fluid adenosine deaminase (ADA) and rapid molecular assays like cartridge-based nucleic acid amplification test (CBNAAT; Xpert MTB/RIF and the newer Xpert-Ultra) to guide early management. ADA, a purine-catabolising enzyme released predominantly by activated T-lymphocytes, rises sharply in the lymphocytic exudate characteristic of TPE. A systematic review of 174 studies (27 009 patients) demonstrated pooled sensitivity and specificity of 92% and 90% respectively, with optimal discriminatory thresholds clustering around 40 IU/L [3]. Indian studies report comparable accuracy; one prospective series recorded 95% sensitivity and 80% specificity using the same cut-off [4]. Nevertheless, ADA levels overlap with those seen in empyema, malignancy and rheumatoid pleurisy and borderline values (40-70 IU/L) pose particular diagnostic dilemmas, prompting calls to integrate ADA with clinical, cytological or biochemical indices for better discrimination [5, 6]. CBNAAT detects *M. tuberculosis* DNA and rifampicin resistance within two hours, bypassing culture requirements. While highly sensitive in sputum-positive pulmonary disease, its performance in pleural fluid has been disappointing. A meta-analysis of 45 studies reported a pooled sensitivity of only 52% against culture, though specificity exceeded 98% [7]. Indian cohorts echo these findings; Chakraborty *et al.* observed sensitivity of 13% yet 100% specificity in biopsy-proven TPE [8], whereas another rural study found 16.6% sensitivity with perfect specificity [9]. The newer Xpert-Ultra, with a ten-fold lower limit of detection, improves yield modestly; a comparative meta-analysis involving 74 studies showed Ultra sensitivity of 68% versus 52% for Xpert, without major loss of specificity [7]. Nevertheless, both assays remain insufficient as stand-alone tests and negative results cannot reliably exclude disease.Given these limitations, international and Indian guidelines advocate a composite approach: in a high-prevalence setting, a lymphocytic exudate with ADA > 40 IU/L or a positive CBNAAT suffices for presumptive treatment, whereas low ADA (<40 IU/L) or inconclusive molecular tests warrant pleural biopsy or thoracoscopy [2, 10]. Emerging algorithms also explore combining ADA with ADA/LDH ratios, interferon-γ assays or ultrasound-guided biopsy to enhance diagnostic certaint [11]. Therefore, it is of interest to assess the diagnostic utility of pleural fluid ADA and CBNAAT individually and in combination and to explore the correlation between ADA activity and molecular detection of *M. tuberculosis* in patients with suspected tubercular pleural effusion.

## Materials and Methods:

This cross-sectional, observational study aimed to evaluate the diagnostic performance of Adenosine Deaminase (ADA) and Cartridge-Based Nucleic Acid Amplification Test (CBNAAT) in diagnosing tubercular pleural effusion. Conduct at the Department of Pulmonary Medicine, LN Medical College and JK Hospital, Bhopal, the study spanned 18 months and involved three phases: planning (3 months), participant recruitment and data collection (12 months) and data analysis and report writing (3 months). Ethical approval was obtained from the Institutional Ethical Committee and informed consent was acquired from all participants. The study included 108 adult patients aged 18 to 65 years, diagnosed with tubercular pleural effusion. A non-probability convenience sampling method was used for recruitment. Participants were eligible if they presented with pleural effusion and met the inclusion criteria, which excluded those with transudative pleural effusion, malignancy, or contraindications to thoracocentesis. Clinical data and pleural fluid samples were collected from each participant. The primary aim was to compare the utility of ADA and CBNAAT in diagnosing tubercular pleural effusion. ADA levels above 40 U/L or those below 40 U/L with corresponding symptoms were considered indicative of tuberculosis, while CBNAAT results were analyzed for *Mycobacterium tuberculosis* DNA presence and rifampicin resistance. Data collection involved clinical interviews, medical records and diagnostic tests. Results were entered into a secure electronic database to ensure confidentiality. Data quality was ensured through regular audits by the study supervisor and oversight by the ethical committee. The statistical analysis was conducted using Stata 17.0, applying descriptive statistics, correlations and graphical representations such as histograms and ROC curves to assess the diagnostic accuracy of ADA and CBNAAT. The study was self-funded and no external funding or conflicts of interest were reported. Data handling and security were strictly maintained, with paper-based data stored securely and electronic data protected with passwords and regular backups. The study aimed to enhance the understanding of ADA and CBNAAT's role in diagnosing tubercular pleural effusion and contribute to improving diagnostic methods in clinical practice.

## Results:

The demographic and socioeconomic data of 108 participants are detailed in Table 1 (see PDF). Males predominated (65.7%) compared to females (34.3%). The majority of participants (30.5%) were aged 21-30 years, followed by those aged 31-40 years (22.2%). Fewer participants fell into other age groups - 18-20 (8.33%), 41-50 (15.7%), 51-60 (13.9%) and 61-65 (9.3%). Most participants were married (73.1%) and resided in rural areas (53.7%). Based on the Kuppuswamy socioeconomic classification, participants were predominantly in the lower (36.1%) and upper-lower (24.1%) classes, with only a small proportion in the upper-middle (13.0%) and upper (3.7%) classes (Table 1 - see PDF). The clinical presentation and symptom duration of participants are summarized in Table 2 (see PDF). The most common presenting complaints were cough (86.1%), shortness of breath (82.4%), fever (80.6%) and chest pain (78.7%). Sputum production and weight loss were noted in 67.6% and 64.8% of cases, respectively and none reported hemoptysis. Nearly half of the participants (48.1%) experienced symptoms for 1-3 months, while 29.6% had symptoms for less than one month. A smaller proportion reported symptoms lasting 4-6 months (18.5%) or 7-9 months (3.7%) (Table 2 - see PDF). Personal habits and clinical examination findings are shown in Table 3 (see PDF). Smoking was reported by 35.2% of participants, alcohol consumption by 34.3% and tobacco chewing by 13.9%. On clinical examination, the mean pulse rate was 92 ± 13 bpm, systolic blood pressure 112 ± 12 mmHg; diastolic blood pressure 76 ± 6 mmHg, oxygen saturation 95 ± 1% and respiratory rate 17 ± 2 breaths/min, indicating overall stable baseline parameters (Table 3 - see PDF). Clinical diagnoses and pleural fluid analysis findings are presented in Table 4 (see PDF). Right-sided tubercular pleural effusion was most common (51.9%), followed by left-sided (45.4%) and bilateral involvement (2.78%). Pleural fluid cytology showed a predominance of mononuclear cells (82 ± 10.3%) over polymorphonuclear cells (17 ± 10.3%). The mean pleural fluid sugar, protein and LDH levels were 70 ± 25.3 mg/dL, 5.21 ± 0.88 mg/dL and 505 ± 325 IU/L respectively, consistent with an exudative, inflammatory process (Table 4 - see PDF).The CBNAAT results and ADA levels are summarized in Table 5 (see PDF). Of the 108 participants, 69.4% (n = 75) tested negative and 30.6% (n = 33) tested positive for CBNAAT. The mean ADA level among CBNAAT-negative participants was 64.9 ± 12.69 IU/L, whereas those testing positive had higher mean levels (79.2 ± 10.81 IU/L). Overall, 91.7% of participants had ADA ≥ 40 IU/L, suggesting a high likelihood of tubercular etiology (Table 5 - see PDF).

## Discussion:

The demographic profile of the present study (n=108) demonstrates a male predominance of 65.7%, closely aligning with other high-tuberculosis-burden settings. In the series by Bielsa *et al.* 70% of patients with tubercular pleural effusion were male, with a median age of 33 years (interquartile range 25-45) [12]. An observational study from Bangladesh similarly found 62% male predominance and noted that the 31-40 year age group comprised 36.3% of cases [13]. In study done by Srinidhi *et al.* [14] out of 100 patients, 80 (80%) are males and 20(20%) females with sex ratio 4:1(M: F). The male predominance seen in there study. In a rural Indian cohort of 75 patients, males accounted for 70.7% of cases and the 20-29 year group represented 32.1% of male participants [15]. These parallels underscore that tubercular pleural effusion disproportionately affects young adult males in endemic regions. Age distribution in the current study showed the highest burden in the 21-30 year bracket (30.5%), followed by 31-40 years (22.2%) and 41-50 years (15.7%). This mirrors the findings of Bielsa *et al.* where half of patients were younger than 45 years [12] and the Bangladesh study, which also reported peak incidence in the third to fourth decades [13]. Marital status and rural residence data are less frequently reported, but the predominance of married (73.1%) and rural (53.7%) participants suggests that socioeconomic and access factors may influence presentation and diagnosis. Clinical presentation in this study was dominated by cough (86.1%), dyspnea (82.4%), fever (80.6%) and chest pain (78.7%). Bielsa *et al.* reported cough in ~70% and chest pain in ~70% of TPE cases [12]. The Bangladeshi cohort found cough in 89.6%, chest pain in 63.2% and fever in 89.6%^2^, while Zhai *et al.* described fever in 88.5% and cough in 68.7% of patients [16]. The higher rates of cough and breathlessness here may reflect variations in healthcare-seeking behavior or disease severity at presentation. Regarding CBNAAT and ADA correlations, the present study's CBNAAT positivity of 30.6% with mean ADA levels of 79.2 IU/L in CBNAAT-positive versus 64.9 IU/L in CBNAAT-negative cases parallels Chakraborty *et al.* who observed 32% CBNAAT positivity with mean ADA 61.7 IU/L, finding a significant association between higher ADA and CBNAAT positivity (p=0.001) [17]. Similarly, in an observational series by Seal *et al.* 75% of patients with ADA >40 IU/L were CBNAAT-positive, while none with ADA ≤40 IU/L tested positive, yielding 100% specificity for the higher ADA cut off. These concordant findings reinforce ADA's value as a surrogate marker for molecular detection of *M. tuberculosis* in pleural fluid.

## Conclusion:

Data shows that tubercular pleural effusion predominantly affects young, economically disadvantaged males from rural areas, often presenting early with cough, dyspnea and fever and chest pain. CBNAAT exhibited high specificity but limited sensitivity, whereas ADA ≥40 IU/L showed robust sensitivity, with higher ADA levels correlating with CBNAAT positivity. Combining ADA measurement with CBNAAT enhances diagnostic accuracy, reducing reliance on invasive procedures and facilitating timely treatment in resource-limited settings.
